# Supplementary figures and images for: Differences in Aroma Profile of Cabernet Sauvignon Grapes and Wines from Four Plots in Jieshi Mountain Region of Eastern China
Source: Foods. 2023 Jul 11;12(14):2668. doi: 10.3390/foods12142668 (PMC10378549; doi:10.3390/foods12142668)

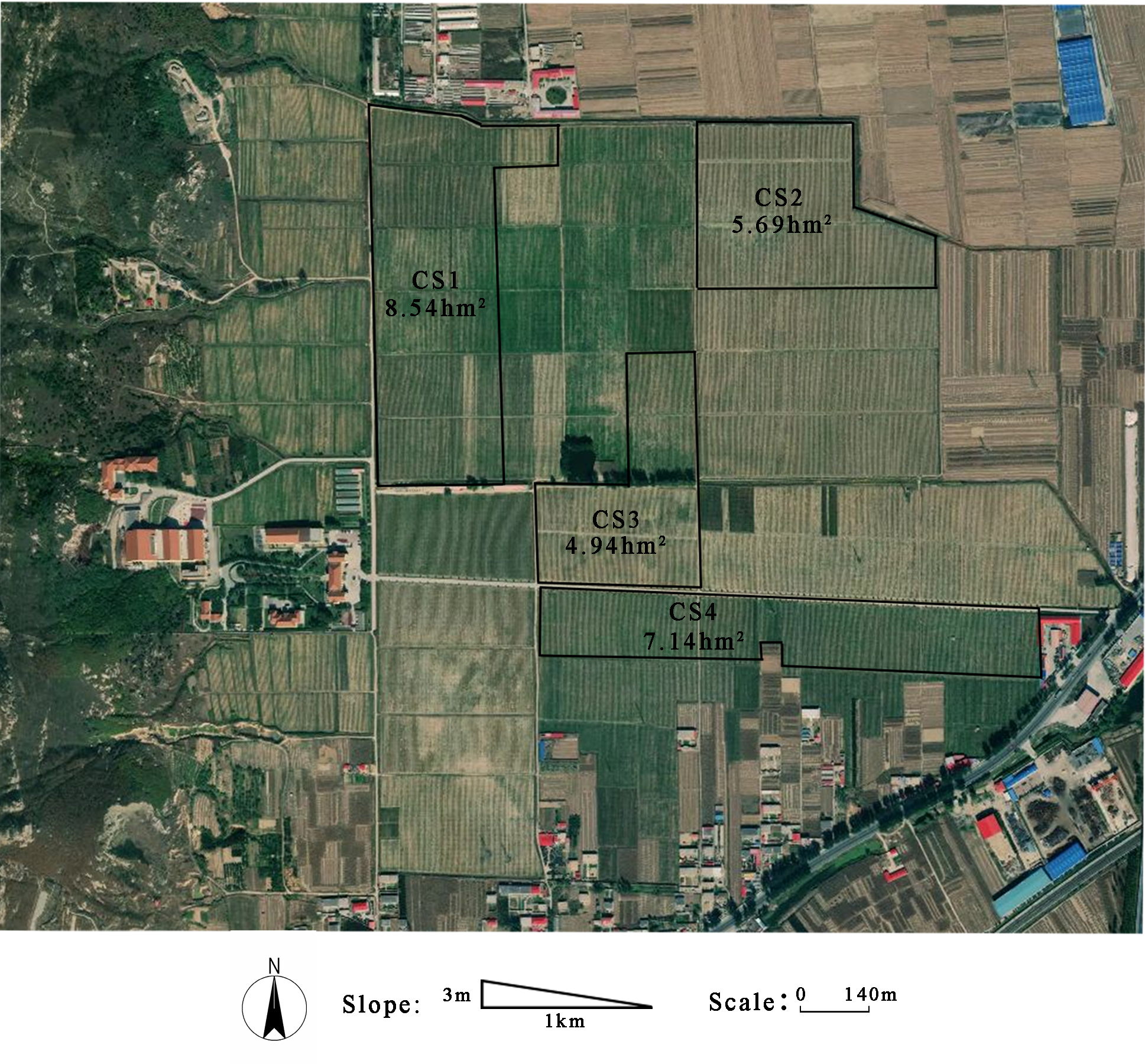

Supplement: Supplementary file 1 [file foods-12-02668-s001.zip › Figure S1 Map of four plots.png]
